# Detection and accurate identification of *Mycobacterium* species by flow injection tandem mass spectrometry (FIA-MS/MS) analysis of mycolic acids

**DOI:** 10.1038/s41598-025-96867-x

**Published:** 2025-04-16

**Authors:** Szewczyk Rafał, Druszczynska Magdalena, Majewski Karol, Szulc Bartłomiej, Kowalski Konrad

**Affiliations:** 1 DiMedical Clinical Medicine Center, DiMedical Sp. z o. o, Zeromskiego 52, 90-626 Lodz, Poland; 2 Department of Immunology and Infectious Biology, Institute of Microbiology, Biotechnology and Immunology, Faculty of Biology and Environmental Protection, University of Lodz, Banacha 12/16, 90-237 Lodz, Poland; 3 LabExperts Sp. z o. o, Sokola 14, 93-519 Lodz, Poland; 4 Medical Department of Bioenergetics and Physiology of Exercise, Faculty of Health Sciences, Medical University of Gdansk, Debinki 1, 80-211 Gdansk, Poland

**Keywords:** *Mycobacterium*, Tuberculosis, Chemotaxonomy, Mycolic acids, Flow injection tandem mass spectrometry (FIA/MS-MS), Innovative diagnostic methods, Tuberculosis, Diagnostic markers, Mass spectrometry, Medical and clinical diagnostics, Clinical microbiology, Infectious-disease diagnostics

## Abstract

Mycolic acids, long-chain α-alkyl, β-hydroxy fatty acids, are characteristic for the genus *Mycobacterium* and play a critical role in the structural integrity and pathogenicity of mycobacterial cell walls. The unique structural diversity of mycolic acids among various *Mycobacterium* species offers a reliable biomarker for taxonomic differentiation. In this study, we applied flow injection analysis coupled with tandem mass spectrometry (FIA-MS/MS) for a comprehensive profiling of mycolic acids. Our findings demonstrate that the structural diversity of mycolic acids can be effectively utilized to enhance the accuracy, sensitivity, and scalability of mycobacterial identification methods. This technique has a broad applications for the rapid identification of mycobacterial pathogens and determination of drug resistance. Particularly in clinical diagnostics it may lead to improvement of patient management and treatment outcomes. Additionally, the method holds promise for epidemiological surveillance, enabling the tracking of mycobacterial outbreaks, as well as applications in environmental microbiology for the detection and monitoring of mycobacteria in diverse ecosystems.

**Supplementary Information:**

The online version contains supplementary material available at 10.1038/s41598-025-96867-x.

## Introduction

The genus *Mycobacterium* encompasses a diverse range of species, including significant human pathogens such as *Mycobacterium tuberculosis*, the etiological agent of tuberculosis (TB), and *Mycobacterium leprae*, which causes leprosy^1^. This genus also includes numerous non-tuberculous mycobacteria (NTM), many of which are increasingly recognized as emerging pathogens, particularly in immunocompromised individuals^2^. The phenotypic and genetic diversity within this genus poses substantial challenges for species identification and classification. Mycobacteria possess complex, lipid-rich cell walls that play a crucial role in their pathogenicity, immune evasion, and resistance to antimicrobial treatments^1^. Among the key components of these cell walls are mycolic acids, which exhibit considerable variability between species in terms of chain length, degree of unsaturation, and functional group modifications^3^. These structural differences have been widely investigated as potential chemotaxonomic markers for distinguishing mycobacterial species. Precise differentiation of mycobacteria is critical for effective clinical diagnostics, epidemiological studies, and the development of public health strategies. Treatment regimens and disease outcomes vary significantly across species, necessitating accurate identification to guide appropriate therapeutic interventions. Additionally, the rise in antibiotic resistance among *Mycobacterium* species further underscores the need for robust diagnostic tools to enable targeted treatments. Advances in lipidomic and mass spectrometry technologies have enhanced the resolution of mycolic acid profiling, offering a promising avenue for improving the identification and characterization of mycobacterial species.

Traditional approaches to the identification of mycobacteria include culture-based methods, which involve growing the bacteria in specialized media, followed by biochemical tests and morphological characterization^4,5^. However, these techniques are laborious, time-consuming, and often yield results after several weeks due to the slow-growing nature of many mycobacterial species. Moreover, certain species exhibit overlapping biochemical profiles, making it difficult to distinguish them with these methods alone. Molecular techniques, such as polymerase chain reaction (PCR) and 16S rRNA gene sequencing, have improved the accuracy of identification, but these approaches also have limitations, including the need for specialized equipment and potential issues with genetic variability within species^4^.

In light of these challenges, lipid profiling has emerged as a complementary method for mycobacterial differentiation. The cell walls of mycobacteria are highly lipid-rich, consisting of complex lipids such as glycolipids, phospholipids, and, most importantly, mycolic acids. Mycolic acids are high-molecular-weight α-alkyl, β-hydroxy fatty acids that are unique to the genus *Mycobacterium* and play a key role in the structural integrity and impermeability of the mycobacterial cell wall^6^. These molecules confer resistance to desiccation, antibiotics, and host immune responses, making them critical to the survival and pathogenicity of mycobacteria^7^. Importantly, the structure of mycolic acids varies between species, particularly in terms of chain length, degree of unsaturation, and functional group modifications, making them excellent taxonomic markers for species differentiation^8^.

Given their structural variability, mycolic acids have been the focus of several studies aimed at differentiating *Mycobacterium* species. Earlier methods of analyzing mycolic acids involved thin-layer chromatography (TLC) and gas chromatography-mass spectrometry (GC-MS)^9–11^. While these techniques provided valuable insights into mycolic acid composition, they were often limited by the need for derivatization steps, lower sensitivity, and longer analysis times. Recent advancements in analytical techniques, particularly the development of liquid chromatography coupled with tandem mass spectrometry (LC-MS/MS), have revolutionized lipidomics by enabling the rapid and sensitive detection of lipids in complex biological samples without extensive sample preparation^12^. LC-MS/MS offers several advantages over traditional methods for mycolic acid profiling. First, it provides high sensitivity and specificity, allowing for the detection of mycolic acids at very low concentrations, which is particularly valuable in clinical samples where bacterial load may be low. Second, LC-MS/MS enables detailed structural elucidation of mycolic acids by analyzing their fragmentation patterns, which can reveal subtle differences in chain length, saturation, and functional group modifications. This level of detail is critical for distinguishing closely related species, such as those within the *Mycobacterium tuberculosis* complex (MTB) and between pathogenic and non-pathogenic NTMs.

In this study, we apply the FIA-MS/MS technique to profile the mycolic acids of various *Mycobacterium* species, with a focus on differentiating clinically relevant pathogens. By leveraging the unique structural features of mycolic acids, we aim to develop a rapid, accurate, and scalable method for the identification of *Mycobacterium* species. This approach has significant implications for improving diagnostic workflows in clinical microbiology laboratories, as well as for advancing our understanding of mycobacterial taxonomy and pathogenesis.

## Results

### Optimization of sample preparation

An extraction procedure adapted from published protocols was employed, involving successive alkaline hydrolysis in an organic solvent at elevated temperatures, followed by neutralization and liquid-liquid extraction^12^. The original protocol for isolating mycolic acids underwent extensive optimization, incorporating 18 distinct modifications. These focused on critical steps such as cell pretreatment, including the extraction and removal of surface lipids using methanol; adjustments to alkali hydrolysis parameters, including alkali concentrations, reaction temperatures, and incubation times; and improvements to the extraction process, such as optimizing the solvent volumes and extraction durations to maximize yield and efficiency. Each modification was meticulously evaluated for reproducibility and repeatability to ensure reliable and consistent results. Compared to the original protocol, the optimized method demonstrated significantly enhanced sensitivity, robustness, and overall analytical performance.

### Validation of the analytical method

The validation method focused on evaluating all parameters characterizing the entire procedure, including sample preparation and FIA-MS/MS analysis, as a diagnostic method for the identification of *Mycobacterium* species and the potential assessment of their drug resistance. Validation encompassed a comprehensive set of parameters: recovery, sensitivity, limit of detection (LOD), lower limit of quantitation (LLOQ), working range, precision, repeatability, reproducibility, relative diagnostic sensitivity, and diagnostic specificity. Each parameter was rigorously tested to ensure the method’s reliability and applicability in clinical diagnostics. Recovery was assessed to verify the efficiency of the extraction and analytical steps, while sensitivity and LOD were evaluated to confirm the method’s ability to detect low concentrations of mycolic acids. Precision and reproducibility analyses included intra- and inter-assay variability to guarantee consistent performance across different runs, laboratories, and operators. Diagnostic sensitivity and specificity were validated against a library of reference strains and clinical isolates to ensure accurate identification of *Mycobacterium* species and their differentiation from non-mycobacterial samples.

The validated method demonstrated high reliability and robustness, making it suitable for routine clinical diagnostics and research applications. A detailed summary of the validation criteria and performance metrics is presented in Table 1.

**Table 1:** Validation results summary.

Parameter	M. tuberculosis H37Rv ATCC 25618	M. smegmatis ATCC 19420	M. absessus ATCC 23045
Limit of detection (S/N)	0,6*10^1^ CFU (4)	0,2*10^1^ CFU (5)	0,4*10^1^ CFU (4)
Lower limit of quantitation (S/N)	1,6*10^1^ CFU (18)	1,0*10^1^ CFU (20)	1,3*10^1^ CFU (18)
Upper limit of quantitation (R^2^)	5,7*10^7^ CFU (0,999)	1,8*10^7^ CFU (0,997)	3,2*10^7^ CFU (0,995)
Precision	± 1,2%	± 2,9%	± 2,2%
Repeatability	± 2,1%	± 4,5%	± 3,8%
Reproducibility	± 9,3%	± 15,3%	± 12,5%

#### Recovery rates and method performance

Recovery studies were performed concurrently on pooled urine and sputum samples collected from healthy volunteers. These samples were spiked with a commercially available mycolic acid mixture derived from *Mycobacterium tuberculosis* (Sigma-Aldrich) to evaluate the efficiency and accuracy of the extraction and quantification process. Enrichment levels of 1 µg/mL and 10 µg/mL of the mycolic acid mixture were used for the experiments. The recovery rates demonstrated excellent method performance, with values of 104.0% and 95.9% observed for sputum samples and 103.5% and 97.5% for urine samples at the 1 µg/mL and 10 µg/mL enrichment levels, respectively. These results indicate a robust and reliable method capable of efficiently extracting and quantifying mycolic acids across a range of concentrations. The slightly elevated recovery values at the lower enrichment level (1 µg/mL) suggest high sensitivity and minimal matrix interference, making this method particularly suited for detecting low concentrations of mycolic acids in clinical samples. Such consistent recovery rates across different biological matrices highlight the method’s versatility and potential applicability in diverse diagnostic and research settings.

#### Limits of detection, quantitation and working range

Tests were conducted to determine the analytical sensitivity and working range of the method for three selected reference strains of *Mycobacterium: M. tuberculosis* H37Rv ATCC 25618, *M. smegmatis* ATCC 19420, and *M. abscessus* ATCC 23045. Bacterial suspensions of defined densities were systematically diluted to achieve appropriate bacterial counts. In parallel, reduction cultures were performed on solid Löwenstein-Jensen (L-J) medium to confirm the exact number of bacteria. The resulting bacterial pellets were subjected to extraction and FIA-MS/MS analysis of mycolic acids. The working range limits of the method were established based on the XIC intensity for the most prominent mycolic acid in the profile of each selected strain. Detection limits (LOD) and the lower limit of quantification (LLOQ) were determined using the signal-to-noise ratio for the chosen XIC. The upper limit of quantification (ULOQ) was defined as the highest bacterial concentration at which the regression parameter (R²) remained greater than 0.990. As shown in Table 1, the developed method demonstrated exceptional analytical sensitivity, capable of detecting single mycobacterial cells. Additionally, it exhibited a wide working range spanning six orders of magnitude. These results highlight the robustness and reliability of the method for quantifying mycolic acids across diverse concentrations, making it well-suited for both low- and high-density bacterial samples in diagnostic and research applications.

#### Method precision, repeatability, and reproducibility

Precision, repeatability, and reproducibility studies were conducted in parallel on three *Mycobacterium* strains previously selected and used for analytical sensitivity testing. Mycobacterial pellets of 10^6^ to 10^7^ CFU (colony forming units), were subjected to mycolic acid extraction and FIA-MS/MS analysis. The area under the XIC curve for the most prominent mycolic acid signal of each strain was used to calculate these validation parameters. The method’s precision was assessed by performing 10 consecutive analyses of the same sample, while repeatability and reproducibility were evaluated through six independent extractions and FIA-MS/MS analyses conducted daily. Repeatability involved a single analyst performing all procedures, whereas reproducibility included the involvement of two different analysts to simulate variations in laboratory handling. The results, expressed as relative standard deviation (RSD), are summarized in Table 1. The findings demonstrate that the developed method, despite being based on the FIA-MS/MS technique, exhibits exceptional precision, repeatability, and reproducibility, ensuring its reliability for robust diagnostic and research applications.

### Mycolic acids as chemotaxonomic biomarkers in the *Mycobacterium* genus

**Fig. 1:**
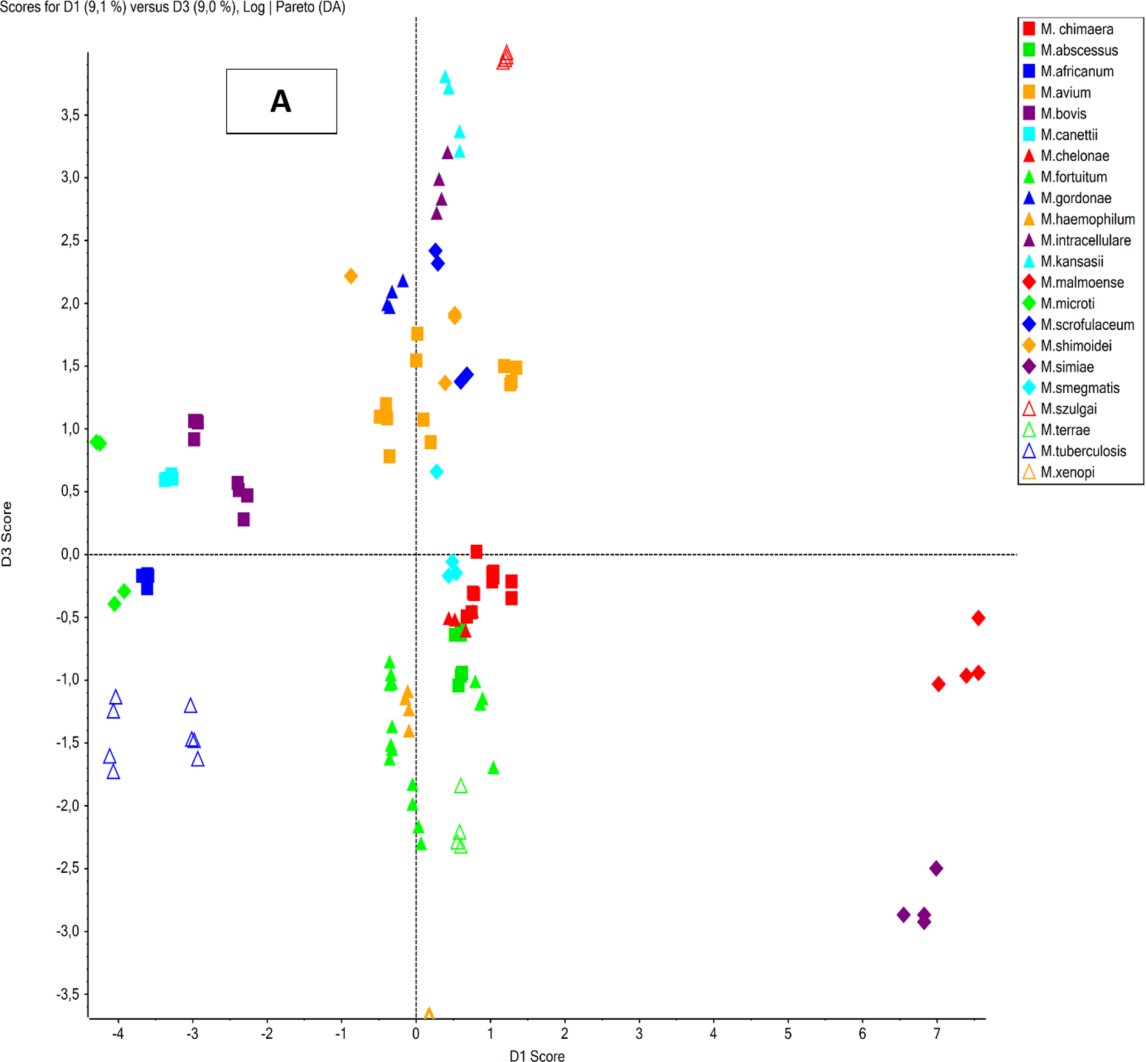

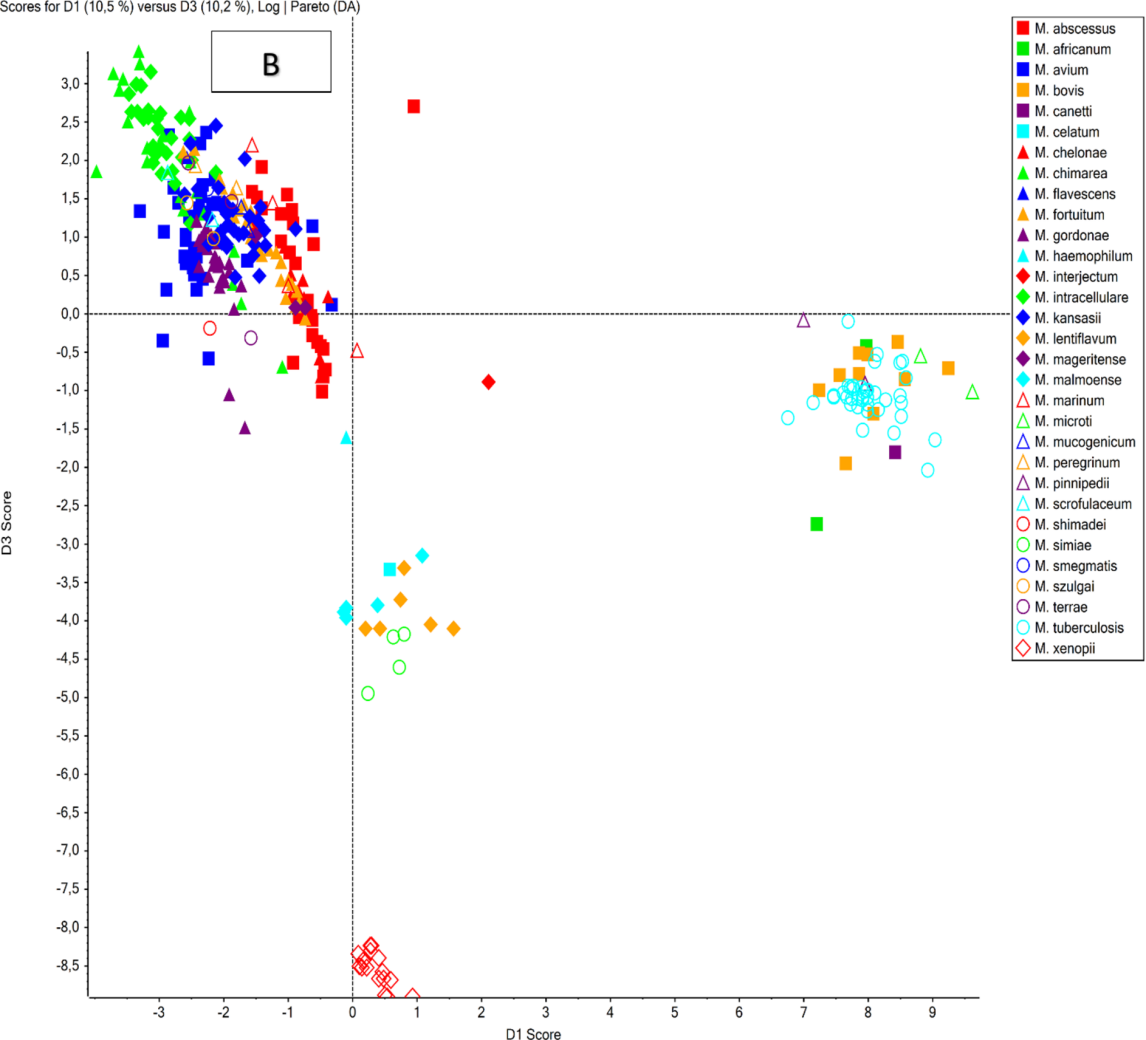
PCA analysis of mycolic acid profiles obtained for (A) only reference strains (32 strains; 4 replicates each) and (B) reference strains and clinical isolates combined (331 strains in total; 1 replicate each).

The PCA analysis of the obtained mycolic acid profiles from the reference strains (Fig. 1a) clearly demonstrates a distinct separation of all analyzed species, based on the simultaneous evaluation of 52 mycolic acids. These findings were further validated by an extended PCA analysis (Fig. 1b), which incorporated the MA profiles of 32 reference strains and 299 clinical isolates. Notably, the analysis of clinical isolates highlights the significant biological diversity of MA profiles, reinforcing their value as chemotaxonomic biomarkers within the *Mycobacterium* genus. Species such as *M. xenopi* and *M. malmoense* are characterized by relatively low heterogeneity and highly conserved MA profiles, reflecting their stable evolutionary traits. Conversely, members of the *Mycobacterium tuberculosis* complex exhibit a mix of high heterogeneity and conserved MA profiles, a pattern distinct from the *Mycobacterium avium-intracellulare* complex group, which is notable for its elevated heterogeneity and reduced profile conservativeness. Other non-tuberculous mycobacterial species display significant diversity among themselves while clustering according to their chemotaxonomic similarities.

**Fig. 2:**
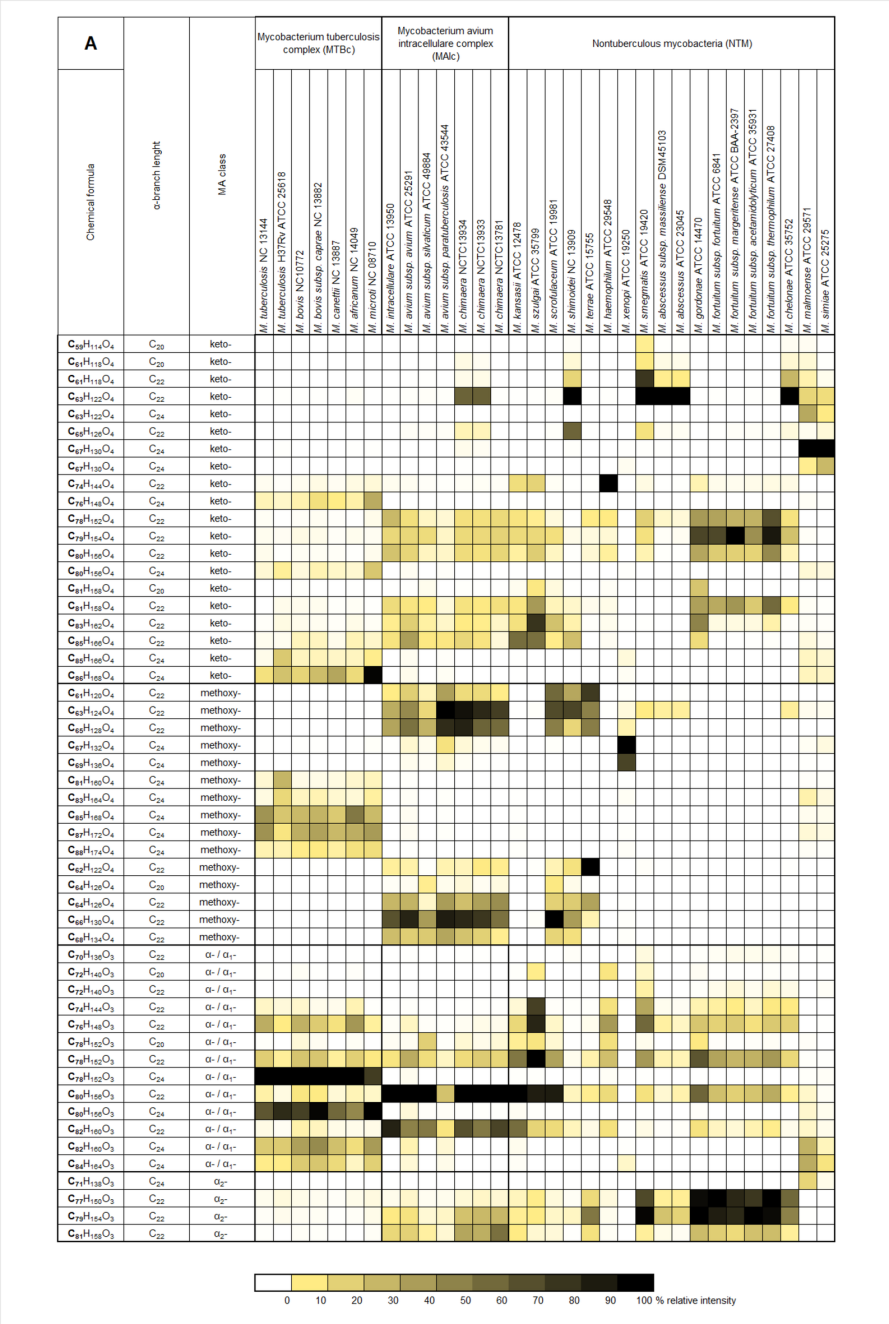

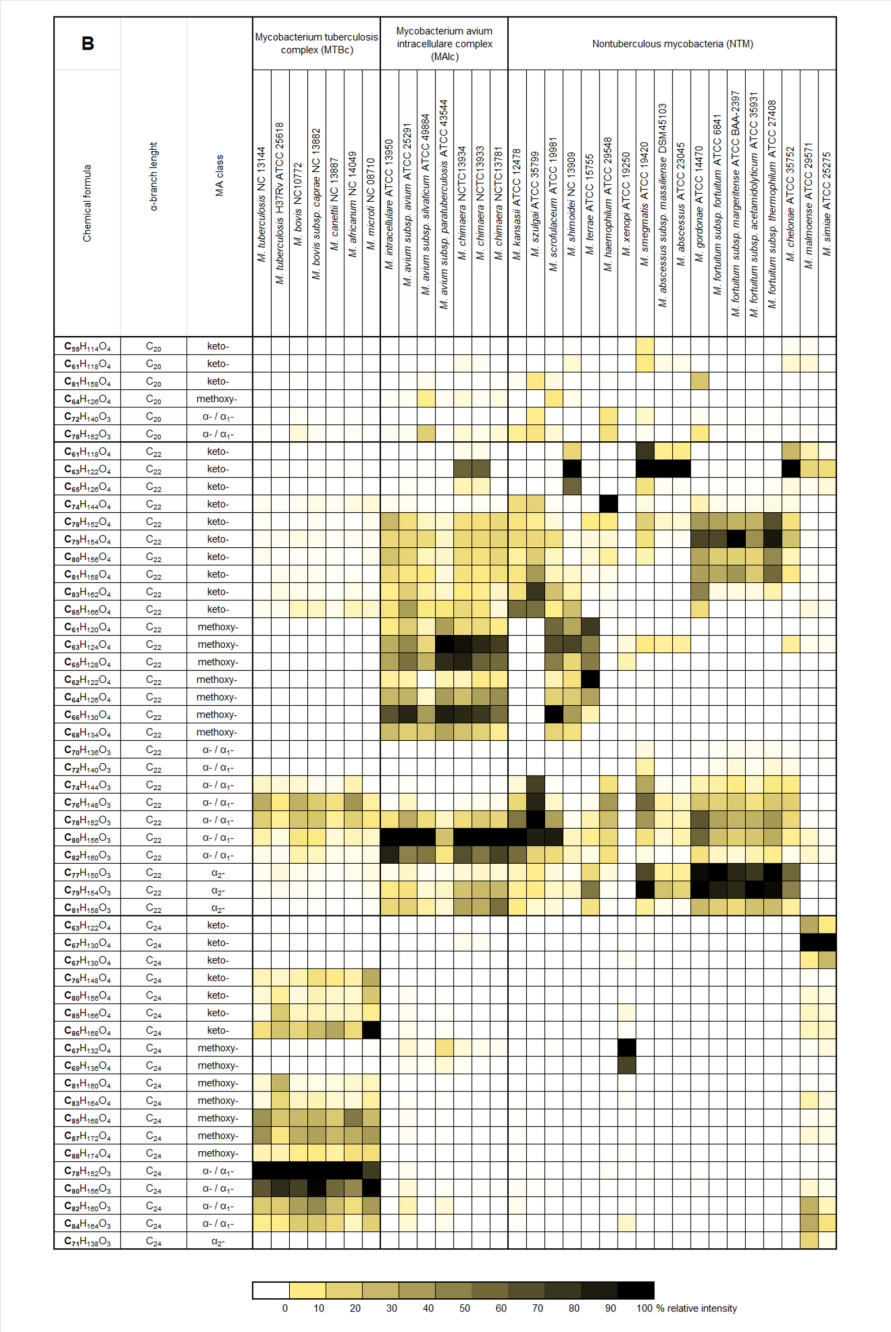
Heatmap of mycolic acid profiles: averaged relative percentage intensity of selected MAs compared to the most intense MA, sorted by (A) MA class and (B) α-alkyl chain length for *Mycobacterium* reference strains.

As shown in Fig. 2a-b, the heat map illustrates the distribution of individual mycolic acids across different *Mycobacterium* groups and strains. Mycolic acid profiles typically consist of MAs from multiple classes present simultaneously, regardless of strain classification. However, an exception is observed with class α_2_-MAs, which are exclusively found in the MAI group and certain NTM strains. Among these, the highest proportions are detected in *M. gordonae*,* M. fortuitum*, and *M. smegmatis.* A much higher level of conservatism is noted in the relationship between α-alkyl chain lengths. Profiles dominated by molecules with the longest α-alkyl chain (C_24_) are characteristic of the MTB group, with only a few exceptions (*M. xenopi*,* M. malmoense*,* and M. simiae)*. Mycolic acids with a C_22_ α-alkyl chain length are the most common across strains, while no strain with a predominant C_20_ α-alkyl chain was observed among the tested reference strains. The identification of pulmonary tuberculosis depends on detecting the distinctive MA profile specific to the MTB group. This profile is characterized by the presence of total mycolic acids ranging from C_74_ to C_88_, with a predominance of MAs containing a C_24_ α-alkyl chain.

### Determination of the drug resistance based on mycolic acids profiles

**Fig. 3:**
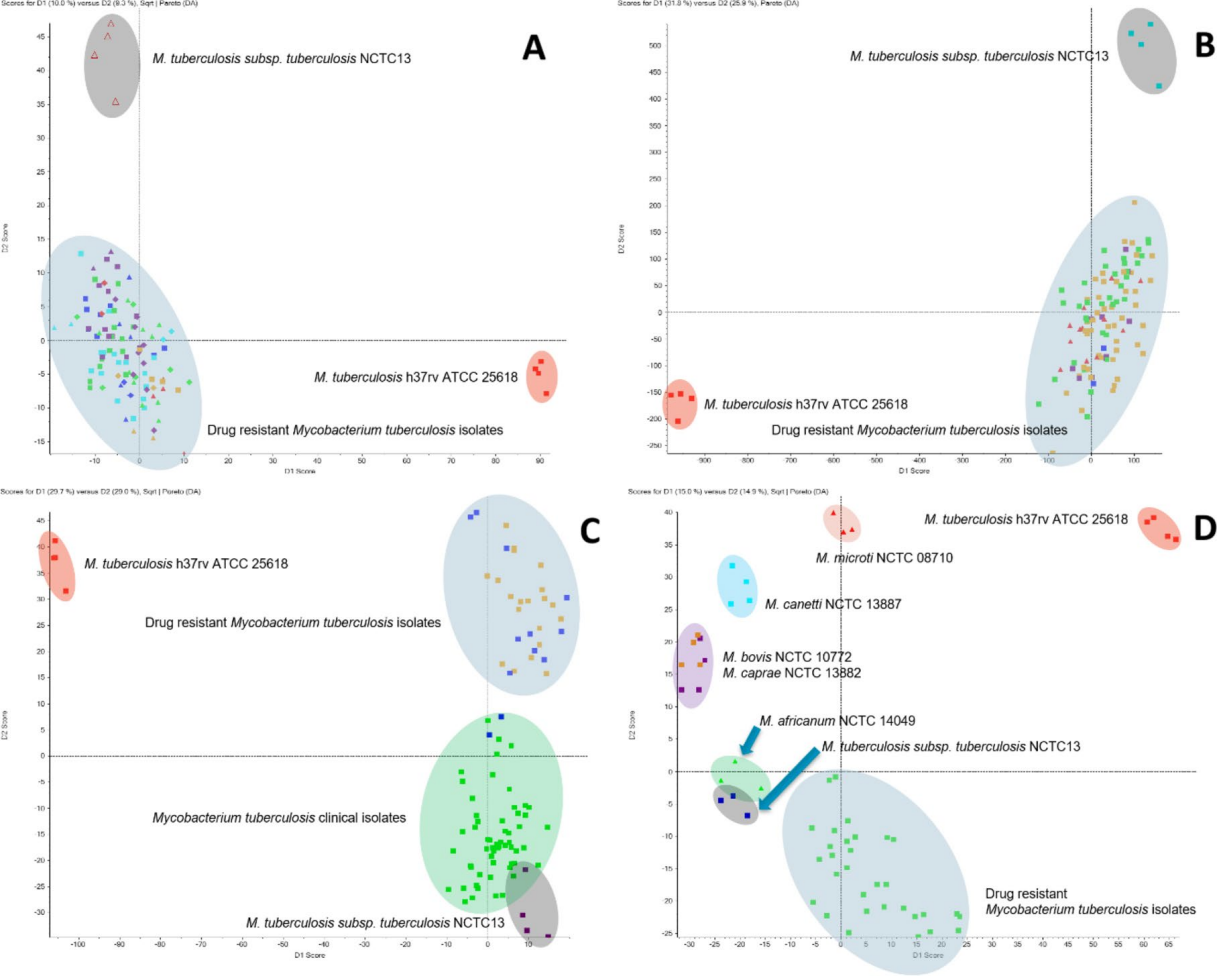
PCA analysis of C_2__4_ α-branch chain (precursor ion) mycolic acids for MTB reference strains with drug-resistant strains (A-B) and MTB reference strains with clinical isolates and drug-resistant strains (C-D), including multidrug-resistant (MDR) and extensively drug-resistant (XDR) tuberculosis, specifying resistance to isoniazid (i), rifampicin, streptomycin (s), ethambutol (e), pyrazinamide (p), amikacin (a), kanamycin (k), ciprofloxacin (c), moxifloxacin (m), and ofloxacin (o).

Based on the culture extraction procedure, MA profiles from MTB and NTM strains with varying drug resistance (see Supplementary Table S1 and S2 online) profiles were examined using both the LC-MS/MS targeted method (MRM) and non-targeted methods (Precursor Ion). In the analysis of MTB strains, neither the targeted method (52 MRM pairs) nor the non-targeted methods yielded specific MA profiles that could precisely define the drug resistance profile (data not shown). However, the obtained profiles did allow for the determination of whether a tested strain is drug-resistant, which may hold significant value as supplementary information alongside species identification. This additional insight could facilitate both immediate clinical decision-making and partial personalization of antibiotic therapy. The observed relationship is effectively illustrated by the PCA analysis presented in Fig. 3, where, despite the inclusion of additional drug-sensitive reference strains, drug-resistant M. tuberculosis isolates form a distinct group within the PCA space. Non-targeted analyses also revealed that, in addition to changes in the intensities of certain MTB MAs used for species identification, there were notable variations in the relative intensities of additional MAs with masses above 1200 m/z. These variations were particularly evident in the C_22_ and C_24_ chains. These additional mycolic acids differentiate drug-resistant mycobacteria more effectively than those traditionally used for genus identification and could be incorporated into targeted methods specifically designed to identify general drug resistance based on the MA profile. In contrast, for NTM strains, no MA profiles were identified that could differentiate between drug-sensitive and drug-resistant strains or determine the drug resistance profile.

### Interference testing and assay selectivity for mycolic acid profiling

To ensure the exclusion of false positives arising from the presence of mycolic acids in bacteria outside the *Mycobacterium* genus, mycolic acid extraction and FIA-MS/MS analysis were conducted on other mycolic acid-containing bacteria within the suborder *Corynebacterinae*. These included *R. equi*,* N. asteroides*,* C. glutamicum*,* D. maris*,* G. bronchialis*,* G. sputi*, and *T. paurometabola.* Remarkably, no false-positive signals were observed for any of these strains, underscoring the high specificity and reliability of the developed assay. This high degree of selectivity highlights the robustness of the method, particularly in differentiating *Mycobacterium* species from other genera that share similar biochemical characteristics. Such specificity is critical for ensuring the accuracy of diagnostic and research applications, reducing the likelihood of misidentification or erroneous conclusions.

### Clinical validation of FIA-MS/MS mycolic acid profiling for the detection of *Mycobacterium* infections

The utility of mycolic acid profiling by FIA-MS/MS for detecting *Mycobacterium* infections was evaluated by analyzing clinical samples from patients with mycobacterial infections (*n* = 36), lower respiratory tract infections not caused by *Mycobacterium* (*n* = 235), and healthy volunteers (*n* = 94). Among patients with mycobacterial infections, a *Mycobacterium*-specific mycolic acid profile was detected in 33 samples using FIA-MS/MS. Of these profiles, 9 were identified as NTM strains, including *M. avium* (*n* = 5), *M. chimaera* (*n* = 2), *M. malmoense* (*n* = 1), and *M. haemophilum* (*n* = 1). The remaining 24 profiles were identified as *M. tuberculosis*. In three samples from patients with tuberculosis, *M. tuberculosis*-specific mycolic acids were not detected. Furthermore, no mycolic acids were detected by FIA-MS/MS in any of the samples from patients with lower respiratory tract infections caused by non-mycobacterial bacteria or in samples from healthy volunteers. The analysis of mycolic acid profiles was performed using M-Typer software, which demonstrated a high degree of similarity between the detected MA profiles and those in the reference library. Notably, in all positive samples, the match between the MA profiles and the library exceeded 90%, underscoring the accuracy and reliability of the method.

In summary, based on the analysis of the results obtained for the clinical samples presented in Table 2, the clinical performance parameters of the FIA-MS/MS test for the detection of *Mycobacterium* infections were calculated. The test demonstrated a specificity of 100%, a sensitivity of 91.6%, a negative predictive value (NPV) of 98.2%, and a positive predictive value (PPV) of 100%.

**Table 2:** Summary of FIA-MS/MS results for clinical samples describing numbers of true positive (TP), true negative (TN), false positive (FP) and false negative (FN) results.

Patients	FIA-MS/MS positive	FIA-MS/MS negative
Mycobacterial infection	33 (TP)	3 (FN)
Non-mycobacterial infection and healthy individuals	0 (FP)	329 (TN)

These results highlight the high reliability of the FIA-MS/MS method, particularly its exceptional ability to exclude false positives due to its perfect specificity. The strong NPV further underscores its utility in confidently ruling out *Mycobacterium* infections in negative cases, while the PPV confirms its precision in accurately identifying positive cases. Such robust diagnostic performance supports the method’s potential as a valuable tool in clinical settings for the rapid and accurate identification of *Mycobacterium* infections, ultimately facilitating timely and targeted patient management.

## Discussion

Mycolic acids are a unique class of chemical molecules that largely define the structure and physiology of the cell wall of mycobacteria. This fact, combined with their physicochemical properties, such as molecular size, conservative structure and high resistance to environmental conditions, make them excellent biomarkers for the presence of mycobacteria in a variety of biological samples. The development of liquid chromatographic techniques in the 1990s allowed the development of a reference method for the classification of mycobacteria based on the profile of bromophenacyl esters of mycolic acids^13^. However, this method and its subsequent modifications did not have sufficient analytical sensitivity for use in the detection of mycobacteria directly from test material^14^. Nowadays, the development of advanced liquid chromatography and/or mass spectrometry techniques, including those combined with ion mobility, allows very detailed analysis of mycolic acid profiles in terms of particle size, structure, conformation and class^15–17^ for chemotaxonomic proposes. Still, the detection and differentiation of *Mycobacterium* species based on their mycolic acid profiles presents a compelling alternative to conventional culture-based and molecular techniques. Leveraging liquid chromatography coupled with tandem mass spectrometry (LC-MS/MS) enables rapid, specific, and sensitive identification of *Mycobacterium* species, significantly enhancing diagnostic workflows. This advancement is particularly vital in settings where prompt and accurate identification is critical, such as tuberculosis management.

The diagnostic sensitivity and specificity of the developed FIA-MS/MS method, reported at 92.66% and 100%, underscores its robustness compared to traditional techniques like bacterioscopy, BacTEC culture, and PCR^18–20^. Those parameters positioning it as a reliable tool for routine diagnostic applications. Unlike methods reliant on bacterial growth or genetic markers, FIA-MS/MS capitalizes on the unique structural diversity of mycolic acids, long-chain fatty acids that serve as biomarkers for species differentiation. These lipids provide a stable and direct basis for identification, circumventing limitations like slow growth rates, contamination, or genetic variability associated with culture and molecular methods.

While culture methods and PCR often exhibit lower sensitivity for certain non-tuberculous mycobacteria, the FIA-MS/MS approach has demonstrated high specificity in identifying species such as *M. avium* and *M. chimaera.* This capability is particularly advantageous for detecting both high-abundance and low-abundance species, including those that are challenging to identify using traditional approaches. By addressing these gaps, FIA-MS/MS strengthens diagnostic precision, especially in complex clinical cases. For instance, recent studies have shown that FIA-MS/MS can reliably differentiate pathogenic NTMs from environmental contaminants, thus enabling targeted therapeutic interventions and reducing misdiagnoses^16^.

The adaptability of FIA-MS/MS is evident in its application to diverse clinical samples, including sputum, bronchoaspirates, pleural fluids, and fine-needle aspiration biopsies. The method’s sensitivity and specificity for diagnosing extrapulmonary MTB and NTM infections highlight its suitability for complex and varied diagnostic needs. This adaptability broadens its clinical utility, ensuring accurate identification even in non-traditional sample types. For example, Fatima et al. (2024) demonstrated that coupling advanced chromatographic methods with LC-MS/MS improves the detection of mycolic acids in difficult-to-process specimens, such as cerebrospinal fluid and bone marrow aspirates^17^. Discrepancies between LC-MS/MS and conventional diagnostic methods, such as bacterioscopy, can often be attributed to the inherent limitations of microscopy in detecting non-tuberculous species. Similarly, the lower sensitivity of BacTEC culture and PCR reflects their dependency on bacterial growth and genetic markers, which may not always be sufficiently expressed or amplified. LC-MS/MS circumvents these issues by directly targeting the intrinsic lipid composition of bacteria, making it a more reliable diagnostic tool. Moreover, the incorporation of cyclic ion mobility-mass spectrometry has further enhanced the specificity of FIA-MS/MS in distinguishing between structurally similar mycolic acid isomers^16^.

The rapid identification capabilities of LC-MS/MS have far-reaching implications for public health. In tuberculosis management, faster diagnosis facilitates earlier and more targeted treatment interventions, potentially reducing transmission and improving patient outcomes. Furthermore, the method’s ability to differentiate NTMs is critical for personalized medicine, as these infections often require distinct therapeutic strategies. Liu et al. (2023) reported that ion mobility mass spectrometry could be used to study lipid variations between drug-resistant and sensitive strains of *M. tuberculosis*, offering insights into mechanisms of resistance and aiding in the development of tailored treatments^21^. Our findings show that the FIA-MS/MS method, despite not yielding profiles precise enough to define the complete drug resistance spectrum, can differentiate drug-resistant *M. tuberculosis* strains from sensitive ones. This capability, demonstrated in PCA analysis, underscores the method’s utility as a complementary diagnostic tool aiding species identification and resistance profiling. The detection of distinct mycolic acid profiles, including variations in C_22_ and C_24_ chains and additional mycolic acids with masses above 1200 m/z, suggests these molecules could serve as markers for resistance detection. In the context of drug susceptibility, this study highlights a critical application of mycolic acid profiling in identifying general resistance patterns in *M. tuberculosis* strains. While the method cannot yet pinpoint specific resistance mechanisms or profiles for individual drugs, its ability to broadly categorize strains as drug-resistant or susceptible has significant implications for clinical decision-making. This insight is particularly valuable in high-burden or resource-limited settings, where rapid and reliable identification of resistance is critical for initiating effective treatment. Early identification of drug resistance can reduce delays in tailoring therapy, limiting disease progression and minimizing transmission risk. Furthermore, our findings provide evidence that non-targeted analyses can uncover additional mycolic acids associated with resistance, particularly in high-molecular-weight fractions. These markers could form the basis for developing new diagnostic assays focused on resistance profiling, complementing existing molecular methods. Future work could refine these markers and validate their use across diverse *M. tuberculosis* strains to improve the specificity and sensitivity of LC-MS/MS in resistance detection. For non-tuberculous mycobacteria our results demonstrate the limitations of mycolic acid profiling in differentiating drug-sensitive and drug-resistant strains. This reflects the inherent diversity and metabolic complexity within non-tuberculous mycobacteria species which pose challenges for both molecular and lipid-based diagnostic methods. However, the specificity of FIA-MS/MS for non-tuberculous mycobacteria identification highlights its potential utility in clinical diagnostics.

From a research perspective, profiling mycolic acids provides new opportunities for studying their structural biology and role in pathogenesis. Insights gained from this lipid-focused analysis could uncover biomarkers for virulence, drug resistance, or environmental persistence, contributing to the development of novel therapeutic or diagnostic targets. For instance, Kumar et al. (2024) explored the use of thin-layer chromatography as a complementary technique to enhance mycolic acid detection in environmental samples, revealing potential applications in epidemiological studies and environmental monitoring of mycobacterial pathogens^22^. Despite its high diagnostic sensitivity, challenges remain in enhancing LC-MS/MS’s capabilities for detecting low-abundance mycobacteria in complex biological matrices. Optimizing sample preparation protocols, such as lipid extraction and enrichment methods, could further improve sensitivity. Additionally, validating the approach across larger, more diverse cohorts would strengthen its clinical applicability. Efforts to improve cost-effectiveness and feasibility in resource-limited settings are critical for the widespread adoption of LC-MS/MS-based diagnostics. Simplified workflows and portable instrumentation, such as miniaturized mass spectrometers, could facilitate implementation in these settings, where rapid and accurate diagnostics are most urgently needed^15^.

LC-MS/MS-based profiling of mycolic acids is a transformative tool for identifying and differentiating *Mycobacterium* species. Its high sensitivity, coupled with the ability to profile structural lipid diversity, positions it as a rapid and scalable diagnostic alternative to conventional methods. Future efforts should prioritize enhancing the method’s sensitivity for low-abundance mycobacteria and expanding its applications in environmental and clinical microbiology. Undoubtedly, LC-MS/MS has the potential to revolutionize diagnostic workflows, improve patient outcomes, and broaden research opportunities in microbial pathogenesis.

## Materials and methods

### Strains and culture conditions

Thirty-two reference strains of *Mycobacterium* were obtained from the ATCC, NCTC, and DSMZ collections (Table 3). Additionally, 299 clinical *Mycobacterium* isolates, primarily recovered from respiratory specimens (e.g., sputum and bronchoaspirates), were cultured under standard laboratory conditions (Table 3). These clinical isolates were collected at the Fryda Laboratory of the Regional Specialized Hospital of Tuberculosis, Lung Diseases, and Rehabilitation in Lodz, Poland and the Institute of Tuberculosis and Lung Diseases in Warsaw, Poland. The mycobacterial cultures were grown in 7H9 Middlebrook broth (Difco Laboratories Ltd., West Molesey, UK), supplemented with 0.05% Tween 80 (Sigma), at 37 °C with constant rotation (125 rpm) until reaching the mid-logarithmic phase. For colony-forming unit (CFU) determination, serial dilutions were plated onto Middlebrook 7H10 agar supplemented with OADC and incubated at 37 °C for up to six weeks. Furthermore, bacterial strains from the *Corynebacterinae* order—including *Corynebacterium glutamicum PCM 1945*,* Rhodococcus equi PCM 559*,* Nocardia asteroides PCM 2138*,* Dietzia maris PCM 2292*,* Gordonia bronchialis PCM 2167*,* Gordonia sputi PCM 2144*,* and Tsukamurella paurometabola PCM 245*3—were purchased from the Polish Collection of Microorganisms (PCM) and cultivated as liquid cultures under specific growth conditions. Careful monitoring of these cultures ensured optimal growth and consistency, which were critical for reproducibility in downstream analyses.

**Table 3:** *Mycobacterium* strains analyzed in the study.

Group	Species	Reference strains	Clinical isolates (*n*)
*Mycobacterium tuberculosis* complex (MTB)	*M. africanum*	*M. africanum* NCTC 14049	1
	*M. bovis*	*M. bovis* NCTC 10772 *M. bovis subsp. caprae* NCTC 13882	6
	*M. bovis BCG*	-	5
	*M. canettii*	*M. canettii* NCTC 13887	-
	*M. microti*	*M. microti* NCTC 08712	-
	*M. pinnipedii*	-	2
	*M. tuberculosis*	*M. tuberculosis subsp. tuberculosis* NCTC 13144 *M. tuberculosis* H37Rv ATCC 25618	40
Non-tuberculosis mycobacteria (NTM)	*M. abscessus*	*M. abscessus* ATCC 23045 *M. abscessus subsp. massiliense* DSM 45103	29
	*M. avium*	*M. avium subsp. avium* ATCC 25291 *M. avium subsp. silvaticum* ATCC 49884 *M. avium subsp. paratuberculosis* ATCC 43544	32
	*M. intracellulare*	*M. intracellulare* ATCC 13950	30
	*M. chimaerae*	*M. chimaera* NCTC 13934 *M. chimaera* NCTC 13933 *M. chimaera* NCTC 13781	19
	*M. celatum*	-	1
	*M. chelonae*	*M. chelonae* ATCC 35752	9
	*M. flavescens*	-	2
	*M. fortuitum*	*M. fortuitum subsp. fortuitum* ATCC 6841 *M. fortuitum subsp. margeritense* ATCC BAA-2397 *M. fortuitum subsp. acetamidolyticum* ATCC 35931 *M. fortuitum subsp. thermophilum* ATCC 27408	22
	*M. gordonae*	*M. gordonae* ATCC 14470	30
	*M. haemophilum*	*M. haemophilum* ATCC 29548	-
	*M. interjectum*	-	1
	*M. kansasii*	*M. kansasii* ATCC 12478	30
	*M. lentiflavum*	-	6
	*M. malmoense*	*M. malmoense* ATCC 29571	6
	*M. marinum*	-	4
	*M. mucogenicum*	-	2
	*M. peregrinum*	-	2
	*M. szulgai*	*M. szulgai* ATCC 35799	1
	*M. scrofulaceum*	*M. scrofulaceum* ATCC 19981	-
	*M. shimoidei*	*M. shimoidei* NC 13909	-
	*M. simiae*	*M. simiae* ATCC 25275	3
	*M. smegmatis*	*M. smegmatis* ATCC 19420	-
	*M. terrae*	*M. terrae* ATCC 15755	2
	*M. xenopi*	*M. xenopi* ATCC 19250	14

### Clinical materials

Clinical samples were collected from 271 patients at the Regional Specialized Hospital of Tuberculosis, Lung Diseases, and Rehabilitation in Lodz, Poland. Among these, 36 patients were diagnosed with lung infections caused by mycobacteria, while 235 had infections caused by other bacteria (Table 4). Additionally, samples from 94 healthy volunteers were included as controls to provide a baseline for comparison. The study was conducted with the approval of the local Bioethics Committee (RNN/785/13/KB) ensuring compliance with ethical standards for the collection and analysis of human samples. Informed consent was obtained from all subjects and/or their legal guardian(s). All experiments were performed in accordance with relevant guidelines and regulations, specifically the recommendations of the Polish Society of Lung Diseases and the National Council of Laboratory Diagnosticians from 2014, as well as the 2018 standards of the Clinical and Laboratory Standards Institute (CLSI), M48 2nd Edition and M24 3rd Edition. The presence of *Mycobacterium* in clinical samples was assessed using a combination of advanced analytical techniques and standard microbiological diagnostic methods. Specifically, flow-injection analysis tandem mass spectrometry (FIA-MS/MS) was employed alongside conventional diagnostic approaches, which included bacterioscopy, culture in BACTEC liquid medium (BD Medical, USA), and the MTB-RIF genetic testing method (Cepheid, USA). All diagnostic results were independently verified to ensure accuracy and reliability. Additionally, the Hain GenoType Mycobacterium CM/AS molecular assay (Hain Lifescience, Germany) was utilized to differentiate between *M. tuberculosis* complex (MTB) and non-tuberculous mycobacteria (NTM). Samples from healthy volunteers underwent identical analyses to confirm the absence of *Mycobacterium* and to validate the specificity of the applied diagnostic methods. A summary of the number of volunteers in the study groups, alongside the types of clinical materials analyzed, is provided in Table 4.

**Table 4:** Summary of the study groups.

	Patients with lung infections (*n*)		Healthy individuals (*n*)
Clinical material	Mycobacterial infection	Non-mycobacterial infection	
Sputum	28	142	94
Bronchoaspirate	7	72	-
Lung biopsy fluid	-	1	-
Pleural fluids	-	15	-
Bronchial catheter fluids	-	5	-
Urine	1	-	-
**Total**	**36**	**235**	**94**

### Sample Preparation

Mycolic acid extraction was performed using the M-Typer Reagent Kit (DiMedical, Lodz, Poland) and comprised three main steps: alkaline hydrolysis at elevated temperature, neutralization, and liquid-liquid extraction. Briefly, a suspension of *Mycobacterium* cells (10⁷ cells/ml) or sputum samples was transferred into a conical glass tube and centrifuged at 3,500 rpm for 10 min at 4 °C. Prior to extraction, sputum samples underwent an additional pretreatment step to liquefy the material using an N-acetylcysteine solution. Only the resulting rinsed pellet, potentially containing *Mycobacterium*, cells was processed in the subsequent mycolic acid extraction steps. Alkaline hydrolysis was performed in a water bath at 90 °C for 60 min. After cooling, neutralization was carried out using a hydrochloric acid solution (18.5% HCl) at a 1:1 volume ratio, resulting in the precipitation of an insoluble salt. The neutralized mixture was then subjected to liquid-liquid extraction at a 2:1 volume ratio, conducted on a rotator-type shaker for 30 min. The organic phase, which settled at the bottom, was carefully collected into a chromatography vial, sealed with a teflon-coated septum cap, and either stored at -80 °C for future use or directly subjected to FIA-MS/MS analysis.

### FIA-MS/MS analysis

The analysis of mycolic acid profiles by flow injection tandem mass spectrometry (FIA-MS/MS) was performed using a QTRAP 6500 + tandem mass spectrometer (Sciex, Toronto, Canada) coupled with an ExionLC AD liquid chromatograph (Sciex, Toronto, Canada). During the study, full compatibility and interchangeability of the M-Typer technology (DiMedical, Lodz, Poland) were demonstrated on a Triple Quad LCMS-8060 system coupled to an LC-20AD chromatograph (Shimadzu, Kyoto, Japan).

The analysis was carried out in negative electrospray ionization (ESI) mode, with the unique signals of mycolic acids (MAs) detected and monitored using the multiple reaction monitoring (MRM) mode. The MRM analysis included monitoring 52 specific transitions, each corresponding to a distinct mycolic acid. These transitions were selected to reflect both the molecular weight of the mycolic acids (Q1 mass) and the length of the α-alkyl chain (Q3 mass). Advanced scheduled MRM methodologies, incorporating MRM grouping and triggering thresholds, ensured sufficient dwell time (> 12 ms) per transition. The panel of 52 MRM transitions covered a broad range of mycolic acid masses, from 887 to 1295 Da, and accounted for variations in α-alkyl chain lengths of C_20_, C_22_, and C_24_. Additionally, the analysis included all major classes of mycolic acids: α-mycolic acids, methoxy-mycolic acids, and keto-mycolic acids (Table 5). As an alternative to targeted MRM analysis, untargeted mass spectrometry methods were employed to explore potential drug resistance profiles. Precursor ion scans detected ions within the range of 850 to 1350 m/z, with fragmentation patterns indicative of α-alkyl chain lengths of C_20_ (339 m/z), C_22_ (367 m/z), and C_24_ (395 m/z), respectively. These scans were conducted in negative ionization mode at a scanning speed of 1000 Da/s, with a defined declustering potential of -150 eV and a collision energy of -76 eV.

**Table 5:** Chemical and mass spectrometry characteristics of mycolic acids used for differentiation of *Mycobacterium* species.

ID	Mycolic acid class	Chemical formula	Pseudomolecular ion [M-H]^−^ [m/z]	α-branch lenght	Fragment ion [M-H]^−^ [m/z]
1	keto-	**C**_**59**_H_114_O_4_	886	C_20_	339
2	keto-	**C**_**61**_H_118_O_4_	914	C_20_	339
3	keto-	**C**_**61**_H_118_O_4_	914	C_22_	367
4	methoxy-	**C**_**61**_H_120_O_4_	916	C_22_	367
5	methoxy-	**C**_**62**_H_122_O_4_	930	C_22_	367
6	keto-	**C**_**63**_H_122_O_4_	942	C_22_	367
7	keto-	**C**_**63**_H_122_O_4_	942	C_24_	395
8	methoxy-	**C**_**63**_H_124_O_4_	944	C_22_	367
9	methoxy-	**C**_**64**_H_126_O_4_	958	C_20_	339
10	methoxy-	**C**_**64**_H_126_O_4_	958	C_22_	367
11	keto-	**C**_**65**_H_126_O_4_	970	C_22_	367
12	keto-	**C**_**67**_H_130_O_4_	970	C_24_	395
13	methoxy-	**C**_**65**_H_128_O_4_	972	C_22_	367
14	methoxy-	**C**_**66**_H_130_O_4_	986	C_22_	367
15	keto-	**C**_**67**_H_130_O_4_	998	C_24_	395
16	methoxy-	**C**_**67**_H_132_O_4_	1000	C_24_	395
17	methoxy-	**C**_**68**_H_134_O_4_	1014	C_22_	367
18	α-	**C**_**70**_H_136_O_3_	1024	C_22_	367
19	methoxy-	**C**_**69**_H_136_O_4_	1028	C_24_	395
20	α_2_-	**C**_**71**_H_138_O_3_	1038	C_24_	395
21	α- / α_1_-	**C**_**72**_H_140_O_3_	1052	C_20_	339
22	α- / α_1_-	**C**_**72**_H_140_O_3_	1052	C_22_	367
23	α- / α_1_-	**C**_**74**_H_144_O_3_	1080	C_22_	367
24	keto-	**C**_**74**_H_144_O_4_	1096	C_22_	367
25	α- / α_1_-	**C**_**76**_H_148_O_3_	1108	C_22_	367
26	α_2_-	**C**_**77**_H_150_O_3_	1122	C_22_	367
27	keto-	**C**_**76**_H_148_O_4_	1124	C_24_	395
28	α- / α_1_-	**C**_**78**_H_152_O_3_	1136	C_20_	339
29	α- / α_1_-	**C**_**78**_H_152_O_3_	1136	C_22_	367
30	α- / α_1_-	**C**_**78**_H_152_O_3_	1136	C_24_	395
31	α_2_-	**C**_**79**_H_154_O_3_	1150	C_22_	367
32	keto-	**C**_**78**_H_152_O_4_	1152	C_22_	367
33	α- / α_1_-	**C**_**80**_H_156_O_3_	1164	C_22_	367
34	α- / α_1_-	**C**_**80**_H_156_O_3_	1164	C_24_	395
35	keto-	**C**_**79**_H_154_O_4_	1166	C_22_	367
36	α_2_-	**C**_**81**_H_158_O_3_	1178	C_22_	367
37	keto-	**C**_**80**_H_156_O_4_	1180	C_22_	367
38	keto-	**C**_**80**_H_156_O_4_	1180	C_24_	395
39	α- / α_1_-	**C**_**82**_H_160_O_3_	1192	C_22_	367
40	α- / α_1_-	**C**_**82**_H_160_O_3_	1192	C_24_	395
41	keto-	**C**_**81**_H_158_O_4_	1194	C_20_	339
42	keto-	**C**_**81**_H_158_O_4_	1194	C_22_	367
43	methoxy-	**C**_**81**_H_160_O_4_	1196	C_24_	395
44	α- / α_1_-	**C**_**84**_H_164_O_3_	1220	C_24_	395
45	keto-	**C**_**83**_H_162_O_4_	1222	C_22_	367
46	methoxy-	**C**_**83**_H_164_O_4_	1222	C_24_	395
47	keto-	**C**_**85**_H_166_O_4_	1250	C_22_	367
48	keto-	**C**_**85**_H_166_O_4_	1250	C_24_	395
49	methoxy-	**C**_**85**_H_168_O_4_	1252	C_24_	395
50	keto-	**C**_**86**_H_168_O_4_	1264	C_24_	395
51	methoxy-	**C**_**87**_H_172_O_4_	1280	C_24_	395
52	methoxy-	**C**_**88**_H_174_O_4_	1294	C_24_	395

**Fig. 4:**
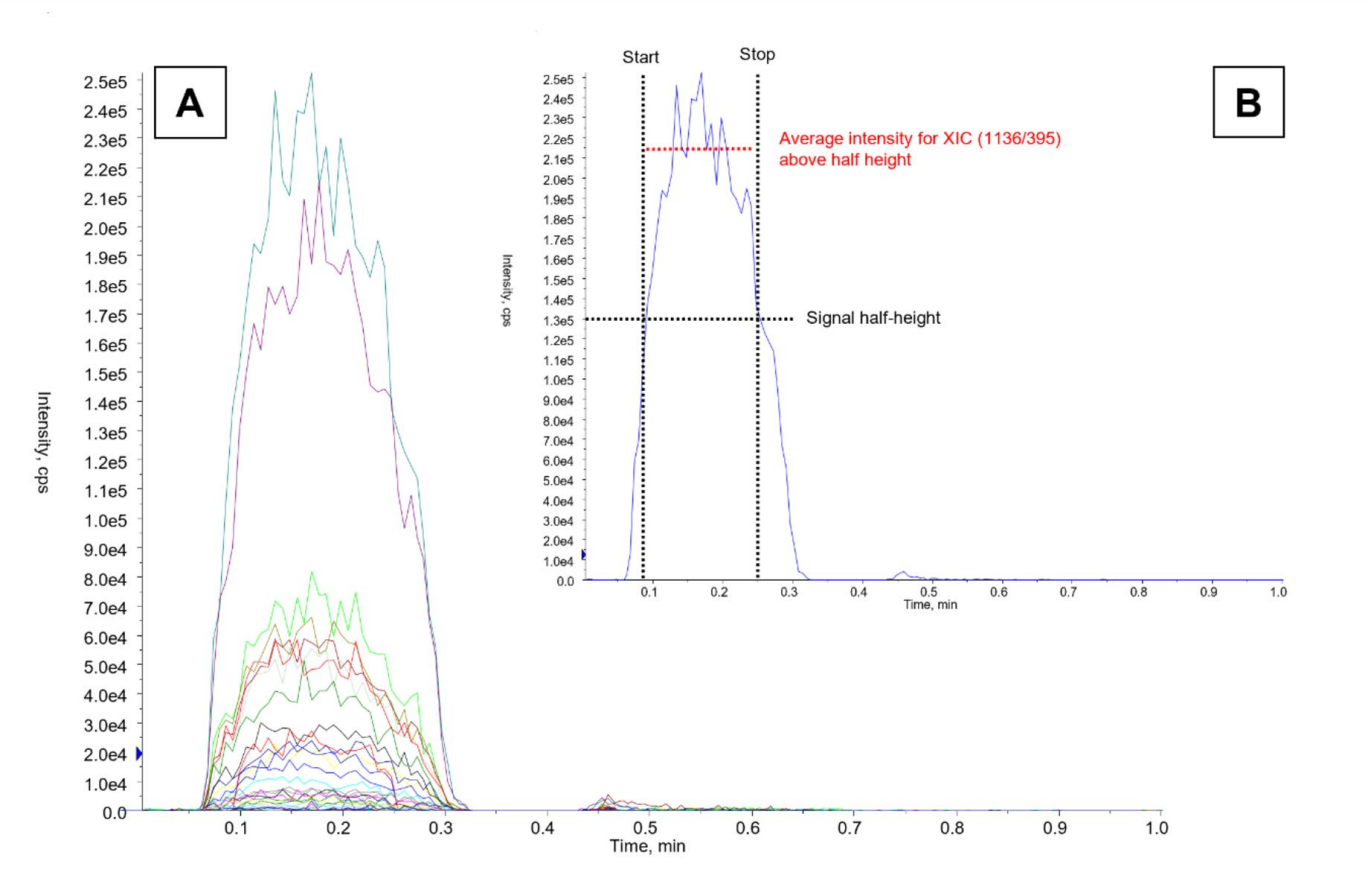
Chromatograms of mycolic acid profiles for Mycobacterium tuberculosis H37Rv. (A) “Hump-like” signal representing extracted ion chromatograms (XIC) for all 52 mycolic acids. (B) XIC (1136/395) with schematic cut-off lines illustrating the signal integration method employed by the M-Typer algorithm.

FIA-MS/MS analysis was performed using reagents (mobile phases and autosampler wash solutions) provided in the M-Typer Reagent Kit (DiMedical, Lodz, Poland). A 50 µL sample of the mycolic acid extract was directly injected into the flow of the ESI-MS/MS system. The gradient flow and mobile phase composition were optimized to achieve the desired “hump-like” shape of the MRM signals while minimizing carryover effects between sequential analyses (Fig. 4). Each analysis was completed within 1 min per sample, with a 30-second interval between consecutive injections.

### Data analysis

The profiles of mycolic acids are represented as the percentage abundance of each mycolic acid relative to the most intense signal among all 52 analyzed. To generate the mycolic acid profile, the extracted ion chromatograms (XICs) for each “hump-like” MA signal obtained through FIA-MS/MS were integrated using M-Typer software. For each XIC, the software calculated the average intensity values that exceeded half the height (between Start – Stop) of the “hump-like” signal after subtracting the baseline noise (Fig. 4). The resulting mean values for all MAs were then compared and normalized against the most intense MA signal from the entire dataset.

The resulting mycolic acid profiles can either be subjected to external statistical analysis or directly analyzed within the M-Typer software framework. To facilitate clinical result interpretation, the M-Typer software compares the generated MA profiles with those stored in its library. This library contains reference data for the mycolic acid profiles characteristic of 331 *Mycobacterium* strains, including both reference strains and clinical isolates, representing 31 distinct species within the *Mycobacterium* genus. Each strain’s data was tested in triplicate and at varying bacterial culture densities (spanning up to four orders of magnitude), resulting in a comprehensive library of more than 4.000 individual mycolic acid profiles. The degree of similarity between an unknown profile and the library profiles is calculated, and a similarity score is assigned to each sample.

Principal component analysis (PCA) was employed to evaluate the discriminatory capacity of the normalized mycolic acid profiles, aiming to distinguish between various strains and groups within the *Mycobacterium* genus. PCA analysis was performed entirely using MarkerView 1.3.1 software (AB Sciex) to explore the potential for group- and species-specific differentiation. Additionally, within the Mycobacterium tuberculosis complex, PCA was applied to assess the feasibility of using mycolic acid profiles for drug susceptibility evaluation.

## Electronic supplementary material

Below is the link to the electronic supplementary material.

Supplementary Material 1

Supplementary Material 2

Supplementary Material 3

## Data Availability

Data can be available after reasonable request to the corresponding author (konrad.kowalski@dimedical.pl). Some of the research protocols presented in the publication are additionally protected by intellectual property rights (EP3978617).
