# Supplementary material for: Detection and accurate identification of Mycobacterium species by flow injection tandem mass spectrometry (FIA-MS/MS) analysis of mycolic acids
Source: Sci Rep. 2025 Apr 16;15:13118. doi: 10.1038/s41598-025-96867-x (PMC12003690; doi:10.1038/s41598-025-96867-x)
Supplement: Supplementary file 1 — Supplementary Material 1 [file 41598_2025_96867_MOESM1_ESM.pdf]

Title: Detection and accurate identification of Mycobacterium species by flow injection tandem mass spectro

Table S1: List of modifications included in the sample preparation optimization.

	KOH (25%, v/v) in methanol solution volume [ml]	Methanol solution [%, v/v]	HCl concentraion [%, v/v]	HCl volume [ml]
Stage I	2	100	18,5	1,5
	2	100	36	1,5
	2	100	18,5	1,8
	2	100	36	1,5
	2	100	18,5	1,8
	2	80	18,5	1,5
	2	80	36	1,5
	2	80	18,5	1,8
	2	80	36	1,5
	2	80	18,5	1,8
	2	50	18,5	1,5
	2	50	36	1,5
	2	50	18,5	1,8
	2	50	36	1,5
	2	50	18,5	1,8
Stage II	2	100	36	1,5
	2	100	36	1,5
	2	100	36	1,5
Stage III	2	100	36	1,5
	2	100	36	1,5
	2	100	36	1,5
	2	100	36	1,5
	2	100	36	1,5

metry (FIA-MS/MS) analysis of mycolic acids

Temperature [C]	Incubation time [min.]	Extraction time [min.]
90	60	60
90	60	60
90	60	60
100	60	60
100	60	60
90	60	60
90	60	60
90	60	60
100	60	60
100	60	60
90	60	60
90	60	60
90	60	60
100	60	60
100	60	60
90	30	60
90	60	60
90	90	60
90	60	10
90	60	20
90	60	30
90	60	45
90	60	60
