# Supplementary material for: Detection and accurate identification of Mycobacterium species by flow injection tandem mass spectrometry (FIA-MS/MS) analysis of mycolic acids
Source: Sci Rep. 2025 Apr 16;15:13118. doi: 10.1038/s41598-025-96867-x (PMC12003690; doi:10.1038/s41598-025-96867-x)
Supplement: Supplementary file 2 — Supplementary Material 2 [file 41598_2025_96867_MOESM2_ESM.pdf]

Title: Detection and accurate identification of Mycobacterium species by flow injection tandem mass spectrometry (FIA-MS/MS) analysis of mycolic acids

Table S2: Detailed drug resistance profile of M. tuberculosis strains subjected to untargeted and targeted FIA-MS/MS analysis.

Drug resistance type	Streptomycin (S)	Isoniazid (I)	Rifampicin (R)	Ethambutol (E)	Pyrazinamide P)	Amikacin (A)	Kanamycin (K)	Ciprofloxacin (C)	Ofloxacin (O)	Moxifloxacin (M)	Number of strains with individual resistance profile (n)	Number of strains with resistance type (n)
non-MDR			•								8	29
	•	•									9	
	•	•		•							2	
		•									7	
			•		•						3	
MDR		•	•								6	41
		•	•	•							1	
	•	•	•								15	
	•	•	•	•							7	
	•	•	•	•	•						2	
	•	•	•		•						10	
pre-XDR		•	•						•		3	17
	•	•	•			•	•				1	
	•	•	•	•					•		1	
	•	•	•	•					•	•	2	
	•	•	•	•	•	•	•	•			3	
	•	•	•	•	•		•				1	
	•	•	•	•	•				•		1	
	•	•	•	•	•				•	•	2	
	•	•	•						•	•	1	
	•	•	•		•	•	•				1	
	•	•	•	•					•		1	
XDR		•	•			•	•	•	•		1	19
		•	•				•		•		1	
	•	•	•	•		•	•	•	•	•	1	
	•	•	•	•				•	•		2	
	•	•	•	•		•	•		•		1	
	•	•	•	•	•	•	•	•	•	•	7	
	•	•	•	•	•	•	•	•	•		1	
	•	•	•	•	•	•	•		•	•	1	
	•	•	•			•	•	•	•		1	
	•	•	•				•		•		1	
	•	•	•		•	•	•	•	•	•	1	
	•	•	•		•		•		•	•	1	
											**Total:**	**106**
