# Supplementary material for: Detection and accurate identification of Mycobacterium species by flow injection tandem mass spectrometry (FIA-MS/MS) analysis of mycolic acids
Source: Sci Rep. 2025 Apr 16;15:13118. doi: 10.1038/s41598-025-96867-x (PMC12003690; doi:10.1038/s41598-025-96867-x)
Supplement: Supplementary file 3 — Supplementary Material 3 [file 41598_2025_96867_MOESM3_ESM.pdf]

Title: Detection and accurate identification of Mycobacterium species by flow injection tandem mass spectrometry (FIA-MS/MS) analysis of mycolic acids

Table S3: Detailed drug resistance profile of mycobacteria other than M. tuberculosis strains subjected to untargeted and targeted FIA-MS/MS analysis.

Strain name	Strain code	Streptomycin	Isoniazid	Rifampicin	Ethambutol	Pyrazinamide	Amikacin	Ofloxacin	Moxifloxacin	Capreomycin	Cycloserine	Ethionamide	Trimethoprim/ sulfamethoxazole	Clofazimine	Erythromycin	Rifabutin	Clarithromycin	Linezolid	Doxycycline
M. abscessus subsp. bolletii	9	nd	nd	nd	nd	nd	S	nd	R	nd	nd	nd	nd	nd	nd	nd	R	S	R
M. abscessus subsp. massiliense	4298	S	R	R	R	nd	nd	R	nd	R	R	nd	R	R	nd	S	nd	nd	nd
M. abscessus subsp. massiliense	652/18	R	R	R	R	nd	S	R	nd	R	R	S	R	R	nd	R	nd	nd	nd
M. abscessus subsp. massiliense	137/16	R	R	R	R	nd	R	R	nd	R	R	nd	R	R	MR	R	nd	nd	nd
M. abscessus subsp. massiliense	4298/18	S	R	R	R	nd	nd	R	nd	R	R	nd	R	R	nd	S	nd	nd	nd
M. abscessuss subsp. abscessus	299/18	R	R	R	R	nd	nd	R	nd	R	R	nd	R	R	nd	R	nd	nd	nd
M. abscessuss subsp. abscessus	6257	R	R	R	R	nd	nd	R	nd	R	R	nd	R	R	nd	R	nd	nd	nd
M. avium	367/18	R	R	R	R	nd	R	R	nd	R	S	nd	S	MS	nd	S	nd	nd	nd
M. avium	321/18	R	R	R	R	nd	R	R	nd	R	S	S	MR	S	nd	S	nd	nd	nd
M. avium	429/18	R	R	R	R	nd	S	R	nd	R	S	nd	MS	S	nd	S	nd	nd	nd
M. avium	1780	nd	nd	nd	nd	nd	nd	nd	nd	nd	nd	nd	nd	nd	nd	nd	S	nd	nd
M. avium	411/18	R	R	R	R	nd	R	R	nd	R	S	nd	MS	MS	nd	MR	nd	nd	nd
M. avium	354/18	nd	nd	nd	nd	nd	nd	nd	nd	nd	nd	nd	nd	nd	nd	nd	S	nd	nd
M. avium	311/18	nd	nd	nd	nd	nd	nd	nd	nd	nd	nd	nd	nd	nd	nd	nd	S	nd	nd
M. avium	500/18	nd	nd	nd	nd	nd	nd	nd	nd	nd	nd	nd	nd	nd	nd	nd	S	nd	nd
M. avium	306/18	nd	R	R	R	nd	S	R	nd	R	S	S	R	S	nd	S	nd	nd	nd
M. avium	297/18	R	R	R	R	nd	nd	S	nd	R	S	S	R	S	nd	S	S	nd	nd
M. avium	305/18	nd	R	R	R	nd	S	S	nd	S	S	S	S	S	nd	S	nd	nd	nd
M. avium	9181	R	R	R	R	nd	nd	R	nd	R	S	nd	MR	S	nd	MR	S	nd	nd
M. avium	274/18	S	R	R	R	nd	nd	R	nd	R	S	S	S	S	nd	S	nd	nd	nd
M. avium	2121	nd	nd	nd	nd	nd	S	S	nd	S	S	S	MS	S	nd	S	nd	nd	nd
M. avium	644/18	nd	nd	nd	nd	nd	R	S	nd	R	S	S	MR	MS	nd	S	S	nd	nd
M. avium	G/8765	R	R	R	R	nd	R	S	nd	S	S	S	S	S	nd	S	nd	nd	nd
M. avium	310/18	R	R	R	R	nd	R	R	nd	R	S	S	MR	S	nd	S	nd	nd	nd
M. avium	396/18	R	R	R	R	nd	nd	nd	nd	nd	nd	nd	nd	nd	nd	nd	nd	nd	nd
M. avium	298/18	R	R	R	R	nd	nd	R	nd	R	S	S	R	S	nd	R	S	nd	nd
M. avium	9050	R	R	R	R	nd	R	R	nd	R	S	S	MR	MR	nd	S	nd	nd	nd
M. avium	414/18	nd	nd	nd	nd	nd	R	R	nd	R	S	nd	MR	S	nd	S	S	nd	nd
M. avium	612/18	nd	nd	nd	nd	nd	nd	nd	nd	nd	nd	nd	nd	nd	nd	nd	S	nd	nd
M. avium	8582	R	R	R	R	nd	S	R	nd	R	S	nd	S	S	nd	MR	S	nd	nd
M. avium	5528	R	R	R	R	nd	S	R	nd	R	S	nd	S	S	nd	S	S	nd	nd
M. avium	474/18	R	R	R	R	nd	S	R	nd	S	S	S	MS	S	nd	MR	nd	nd	nd
M. avium	8257	R	R	R	R	nd	S	R	nd	R	S	nd	S	S	nd	MR	S	nd	nd
M. avium	411	R	R	R	R	nd	R	R	nd	R	S	nd	MS	MS	nd	MR	nd	nd	nd
M. chelonae	9341	S	R	R	R	nd	nd	R	nd	S	R	S	S	R	nd	S	nd	nd	nd

M. chelonae	8181	nd	nd	nd	nd	nd	nd	R	nd	R	R	S	R	R	nd	R	nd	nd	nd
M. chelonae	47	R	R	R	R	nd	S	nd	MS	nd	nd	nd	nd	R	nd	nd	S	S	nd
M. chelonae	54	R	R	R	R	nd	S	nd	MS	nd	nd	nd	nd	R	nd	nd	S	S	nd
M. chelonae	2308	S	R	S	R	nd	nd	R	nd	S	R	S	nd	nd	nd	nd	nd	nd	nd
M. chimaera	4463	R	R	R	R	nd	nd	R	nd	R	S	S	MR	S	nd	S	nd	nd	nd
M. chimaera	300/18	S	R	R	R	nd	R	R	nd	R	R	S	R	S	nd	R	nd	nd	nd
M. chimaera	4338	R	R	R	R	nd	R	R	nd	R	R	S	R	S	nd	S	nd	nd	nd
M. chimaera	7735	R	R	R	S	nd	S	R	nd	R	S	nd	MS	S	nd	S	S	nd	nd
M. chimaera	2795	R	R	R	R	nd	nd	R	nd	R	S	nd	MR	S	nd	S	S	nd	nd
M. chimaera	2648	S	R	R	R	nd	nd	R	nd	S	nd	S	MR	S	nd	S	S	nd	nd
M. chimaera	672/18	R	R	R	R	nd	S	R	nd	R	S	S	R	S	nd	S	nd	nd	nd
M. chimaera	8313	R	R	R	R	nd	S	R	nd	R	R	S	MR	S	nd	S	S	nd	nd
M. chimaera	108	R	R	R	R	nd	R	R	nd	R	R	S	R	S	nd	S	nd	nd	nd
M. chimaera	5382	R	R	R	S	nd	S	R	nd	R	S	nd	MR	S	nd	S	S	nd	nd
M. chimaera	4	R	R	R	R	nd	R	R	nd	R	R	S	R	S	nd	S	nd	nd	nd
M. fortuitum	349/18	R	R	R	R	nd	S	S	nd	S	R	nd	S	R	nd	S	nd	nd	nd
M. fortuitum	897	R	R	R	R	nd	nd	S	nd	S	R	S	S	R	nd	R	nd	nd	nd
M. fortuitum	1080	R	R	R	R	nd	nd	S	nd	S	R	S	S	R	nd	R	nd	nd	nd
M. fortuitum	1151	R	R	R	R	nd	nd	S	nd	S	R	S	S	R	nd	R	nd	nd	nd
M. fortuitum	2011	S	R	R	R	nd	nd	S	nd	S	R	S	R	R	nd	R	nd	nd	nd
M. fortuitum	8907	R	R	R	R	nd	nd	S	nd	S	R	nd	MR	R	nd	R	nd	nd	nd
M. fortuitum	327/18	R	R	R	R	nd	nd	nd	nd	nd	nd	nd	nd	nd	nd	nd	nd	nd	nd
M. fortuitum	325/18	R	R	R	R	nd	nd	nd	nd	nd	nd	nd	nd	nd	nd	nd	nd	nd	nd
M. fortuitum	395/18	R	R	R	R	nd	S	S	nd	S	R	S	R	R	nd	R	nd	nd	nd
M. fortuitum	322/18	R	R	R	R	nd	S	S	nd	S	R	nd	R	R	nd	R	nd	nd	nd
M. fortuitum	326/18	R	R	R	R	nd	S	S	nd	S	R	nd	R	R	nd	R	nd	nd	nd
M. fortuitum	352/18	R	R	R	R	nd	S	S	nd	S	R	S	MS	R	nd	R	nd	nd	nd
M. fortuitum	424/18	R	R	R	R	nd	S	S	nd	S	R	S	R	R	nd	R	nd	nd	nd
M. fortuitum	7977	R	R	R	R	nd	S	S	nd	S	R	nd	R	R	nd	R	nd	nd	nd
M. fortuitum	7202	R	R	R	R	nd	S	S	nd	S	R	S	R	R	nd	R	nd	nd	nd
M. gordonae	1398	S	R	S	S	nd	nd	S	nd	S	S	S	nd	nd	nd	nd	nd	nd	nd
M. gordonae	2441	S	R	S	S	nd	nd	S	nd	S	S	S	nd	nd	nd	nd	nd	nd	nd
M. gordonae	2006	S	R	R	S	nd	nd	S	nd	S	S	S	S	S	nd	S	nd	nd	nd
M. gordonae	625/18	S	R	S	S	nd	S	S	nd	S	S	S	S	S	nd	S	nd	nd	nd
M. gordonae	647/18	R	R	S	S	nd	S	S	nd	S	S	S	nd	nd	nd	nd	nd	nd	nd
M. gordonae	7402	S	R	S	S	nd	nd	S	nd	S	S	S	S	S	nd	S	nd	nd	nd
M. gordonae	1175	S	R	S	S	nd	nd	S	nd	S	S	S	S	S	nd	S	nd	nd	nd
M. gordonae	388	S	R	S	S	nd	nd	S	nd	S	S	nd	S	S	nd	S	nd	nd	nd
M. gordonae	350/18	S	R	S	S	nd	S	S	nd	S	S	S	nd	nd	nd	nd	nd	nd	nd
M. gordonae	236/18	S	R	S	S	nd	nd	S	nd	S	S	nd	S	S	nd	S	nd	nd	nd
M. intracellulare	767	S	R	R	S	nd	nd	S	nd	R	S	S	R	S	nd	S	nd	nd	nd
M. intracellulare	915	S	R	R	R	nd	nd	S	nd	S	R	S	MS	S	nd	S	nd	nd	nd
M. intracellulare	4276	R	R	R	S	nd	nd	R	nd	R	S	nd	R	S	nd	S	nd	nd	nd
M. intracellulare	655/18	nd	nd	nd	nd	nd	nd	nd	nd	nd	nd	nd	nd	nd	nd	nd	S	nd	nd
M. intracellulare	508/18	nd	nd	nd	nd	nd	S	R	nd	S	S	S	MS	S	nd	S	nd	nd	nd
M. intracellulare	1028	S	R	S	S	nd	nd	S	nd	S	S	S	R	S	nd	S	nd	nd	nd
M. intracellulare	3363	S	R	S	S	nd	nd	R	nd	S	S	S	R	S	nd	S	nd	nd	nd
M. intracellulare	5987	R	R	R	R	nd	nd	R	nd	R	S	nd	MR	S	nd	S	nd	nd	nd
M. intracellulare	527/18	R	R	R	R	nd	S	R	nd	R	S	nd	MR	S	nd	S	nd	nd	nd
M. intracellulare	277	S	R	R	S	nd	nd	S	nd	S	S	S	R	MS	nd	S	S	nd	nd

M. intracellulare	2163	S	R	R	R	nd	R	R	nd	S	S	nd	R	S	R	S	nd	nd	nd
M. intracellulare	2632	nd	R	R	S	nd	nd	R	nd	S	S	nd	MS	S	nd	S	nd	nd	nd
M. intracellulare	525	nd	nd	nd	nd	nd	nd	nd	nd	nd	nd	nd	MR	S	nd	S	nd	nd	nd
M. intracellulare	836	R	R	R	S	nd	nd	R	nd	R	R	nd	R	S	nd	S	S	nd	nd
M. intracellulare	835	R	R	R	S	nd	nd	R	nd	R	R	nd	R	S	nd	S	S	nd	nd
M. intracellulare	595/17	nd	nd	nd	nd	nd	nd	R	nd	R	R	S	R	S	nd	S	nd	nd	nd
M. intracellulare	1848	R	R	R	S	nd	nd	R	nd	S	S	S	MR	S	nd	S	nd	nd	nd
M. intracellulare	527/17	R	R	R	R	nd	nd	R	nd	R	S	nd	MS	S	nd	S	nd	nd	nd
M. intracellulare	3210	S	R	S	S	nd	nd	R	nd	S	S	S	R	S	nd	S	nd	nd	nd
M. intracellulare	5639	R	R	R	S	nd	S	R	nd	R	S	nd	R	S	nd	S	S	nd	nd
M. intracellulare	2166	S	R	R	S	nd	R	S	nd	S	S	S	R	S	nd	MR	nd	nd	nd
M. intracellulare	675/18	nd	nd	nd	nd	nd	nd	nd	nd	nd	nd	nd	nd	nd	nd	nd	S	nd	nd
M. intracellulare	4554	R	R	R	S	nd	nd	R	nd	R	S	nd	R	S	nd	S	S	nd	nd
M. intracellulare	8925	S	R	R	R	nd	S	S	nd	S	S	S	MS	MS	nd	S	S	nd	nd
M. intracellulare	2218	nd	nd	nd	nd	nd	S	S	nd	S	S	S	S	S	nd	S	nd	nd	nd
M. kansasii	522/18	S	R	S	S	nd	S	S	nd	S	S	S	S	S	nd	S	nd	nd	nd
M. kansasii	673/18	S	R	S	S	nd	S	S	nd	S	S	S	S	S	nd	S	nd	nd	nd
M. kansasii	394/18	S	R	S	S	nd	S	S	nd	S	S	S	S	S	nd	S	nd	nd	nd
M. kansasii	670/18	S	R	S	S	nd	S	S	nd	S	S	S	S	S	nd	S	nd	nd	nd
M. kansasii	780	nd	nd	nd	nd	nd	S	S	nd	S	S	S	S	S	nd	S	nd	nd	nd
M. kansasii	438/18	nd	nd	nd	nd	nd	S	S	nd	S	S	S	S	S	nd	S	nd	nd	nd
M. kansasii	465/18	S	R	S	S	nd	S	S	nd	S	S	S	S	S	nd	S	nd	nd	nd
M. kansasii	423/18	S	R	S	S	nd	S	S	nd	S	S	S	S	S	nd	S	nd	nd	nd
M. kansasii	5917	nd	nd	nd	nd	nd	MS	S	nd	S	S	S	S	S	S	S	nd	nd	nd
M. kansasii	309/18	S	R	S	S	nd	nd	S	nd	S	S	S	S	S	nd	S	nd	nd	nd
M. kansasii	G432	S	R	S	S	nd	S	S	nd	S	S	S	nd	nd	nd	nd	nd	nd	nd
M. kansasii	6178	S	R	S	S	nd	S	S	nd	S	S	S	nd	S	nd	S	nd	nd	nd
M. kansasii	1448 BK	S	R	S	S	nd	S	S	nd	S	S	S	S	S	nd	S	nd	nd	nd
M. kansasii	368/18	S	R	S	S	nd	S	S	nd	S	S	S	S	S	nd	S	nd	nd	nd
M. kansasii	9255	S	R	S	S	nd	S	S	nd	S	S	S	nd	nd	nd	nd	nd	nd	nd
M. kansasii	8514	S	R	S	S	nd	S	S	nd	S	S	S	S	S	nd	S	nd	nd	nd
M. kansasii	7761	S	R	S	S	nd	S	S	nd	S	S	S	S	S	nd	S	nd	nd	nd
M. lentiflavum	6852	R	R	R	R	nd	R	S	nd	R	S	S	S	S	nd	S	nd	nd	nd
M. mageritense	7451	R	R	R	R	nd	nd	S	nd	S	R	nd	R	R	nd	R	nd	nd	nd
M. mageritense	497	R	R	R	R	nd	S	R	nd	S	R	nd	S	R	nd	R	nd	nd	nd
M. mageritense	580	R	R	R	R	nd	S	S	nd	S	R	S	R	R	nd	R	nd	nd	nd
M. malmoense	646/18	nd	nd	nd	nd	nd	nd	nd	nd	nd	nd	nd	MR	S	nd	S	nd	nd	nd
M. malmoense	642/18	nd	nd	nd	nd	nd	nd	nd	nd	nd	nd	nd	R	S	nd	S	nd	nd	nd
M. simiae	657	nd	R	R	R	nd	nd	R	nd	R	R	nd	R	S	nd	R	nd	nd	nd
M. smegmatis	3679	S	S	S	S	nd	nd	S	nd	S	S	S	S	S	nd	S	nd	nd	nd
M. xenopi	149	S	S	R	S	nd	nd	S	nd	S	S	S	S	S	nd	S	nd	nd	nd
M. xenopi	2397	S	R	S	S	nd	nd	S	nd	S	S	S	S	S	nd	S	nd	nd	nd
M. xenopi	2314	S	R	S	S	nd	nd	S	nd	S	S	S	S	S	nd	S	nd	nd	nd
M. xenopi	8970	S	R	S	S	nd	nd	S	nd	S	S	S	S	S	nd	S	nd	nd	nd
M. xenopi	492	S	S	S	S	nd	nd	S	nd	S	S	S	S	S	nd	S	nd	nd	nd
M. xenopi	1062	S	R	S	S	nd	nd	S	nd	S	S	S	S	S	nd	S	nd	nd	nd
M. xenopi	799	S	R	R	S	nd	nd	S	nd	S	S	S	S	S	nd	S	nd	nd	nd
M. xenopi	7553	S	R	R	S	nd	nd	S	nd	S	S	S	S	S	nd	S	nd	nd	nd
M. xenopi	2466	S	S	S	S	nd	nd	S	nd	S	S	S	S	S	nd	S	nd	nd	nd
M. xenopi	392/18 BK	S	R	S	S	nd	nd	S	nd	S	S	S	S	S	nd	S	nd	nd	nd

M. xenopi	1990	S	S	S	S	nd	nd	S	nd	S	S	S	S	S	nd	S	nd	nd	nd
M. xenopi	442/18	nd	nd	nd	nd	nd	S	S	nd	S	S	S	S	S	nd	S	nd	nd	nd
M. xenopi	318/18	S	S	S	S	nd	S	S	nd	S	S	S	S	S	nd	S	nd	nd	nd
M. xenopi	7785	S	R	R	S	nd	S	S	nd	S	S	S	S	S	nd	S	nd	nd	nd
M. xenopi	7533	S	R	S	S	nd	S	S	nd	S	S	S	S	S	nd	S	nd	nd	nd

Abbreviations:

R - resistant

MR - moderately resistant

MS - moderately susceptible

S - susceptible

nd - no data
